# Longitudinal changes in sleep quality, and their predictors in patients with multiple sclerosis

**DOI:** 10.1038/s41598-025-18693-5

**Published:** 2025-09-26

**Authors:** Habin Yun, Dayoung Seo, Yumin Jin, In-Hye Jang, Lynkyung Choi, Jin-Hee Kim, Wangyoung Shin, Hye-Mi Lee, Hee-Jae Jung, Hyunjin Kim, Young-Min Lim, Eun-Jae Lee

**Affiliations:** 1 University of Ulsan College of Medicine, Seoul, 05505 South Korea; 2 Department of Neurology, Asan Medical Center, University of Ulsan, Seoul, 05505 South Korea

**Keywords:** Multiple sclerosis, Sleep quality, PSQI, Neurologic disability, Longitudinal sleep quality, Medical research, Neurology

## Abstract

Sleep disturbances are common among patients with multiple sclerosis (PwMS), yet their longitudinal course and clinical determinants remain unclear. Here, we aimed to examine sleep quality in PwMS, investigate its association with quality of life, and evaluate its trajectory and clinical predictors over time. Between September 2022 and September 2023, PwMS who had not experienced a recent clinical relapse (within ≤ 2 months) were prospectively recruited. Sleep quality at baseline and after 6–12 months was measured using the Pittsburgh Sleep Quality Index (PSQI). Clinical factors associated with PSQI scores, and their longitudinal trajectories, were examined. A total of 118 patients with MS (median age, 46 years) were enrolled, with 54% identified as poor sleepers (PSQI > 5) at baseline and a comparable prevalence (53%) at follow-up. Poor sleep quality was associated independently with reduced quality of life. While PSQI scores remained generally stable over time in participants without clinical relapses during follow-up (*n* = 114), 23 (20.2%) exhibited a marked deterioration in sleep quality (PSQI score increase ≥ 3). Baseline optic Functional Systems scores (baseline: β = 0.226 (95% confidence interval, 0.021, 1.115), follow-up: β = 0.233, (0.038, 1.311)) and depression (baseline: β = 0.261, (1.031, 5.822), follow-up: β = 0.215, (0.473, 5.935)) independently predicted both baseline and future PSQI scores. In conclusion, poor sleep quality is common and persistent in PwMS, with depression and the severity of optic dysfunction identified as key predictors. These findings emphasize the need for targeted sleep management in MS care.

**Supplementary Information:**

The online version contains supplementary material available at 10.1038/s41598-025-18693-5.

## Introduction

Multiple sclerosis (MS) is a chronic autoimmune disease of the central nervous system (CNS), characterized by immune-mediated demyelination, axonal injury and loss, and subsequent neurodegeneration. Although it most commonly presents in young adults and follows a lifelong trajectory of relapses and progression, its underlying immunopathology, clinical manifestations, and prognosis are variable, resulting in a wide spectrum of neurological impairment^1–5^.

Sleep disturbance and poor sleep quality are common and often under-recognized manifestations in patients with MS (PwMS), affecting more than half of this population^6,7^. These disturbances encompass a range of conditions, including restless leg syndrome (RLS), insomnia, and obstructive sleep apnea (OSA)^8–11^. Poor sleep quality is associated with a more severe disease course^12^ and is linked closely to key MS symptoms such as fatigue^13^ cognitive decline^14^ and reduced health-related quality of life (HRQoL)^7^. Despite its high prevalence and considerable impact, the long-term trajectory of sleep quality in PwMS, along with the key determinants and predictors of poor sleep, remains unclear.

Given the wide spectrum of clinical trajectory and CNS involvement in MS, we hypothesized that sleep quality in PwMS would evolve over the disease course, and that these changes may be influenced by individual differences in brain function. To test these hypotheses, we designed a prospective study to examine longitudinal changes in sleep quality among PwMS, investigate its association with quality of life, and identify clinical variables that determine or predict sleep quality over time.

## Materials and methods

### Patients and study design

This study was a single-center, prospective, observational cohort investigation with a follow-up period of 6–12 months. Between September 2022 and September 2023, participants were recruited from the Neurology Outpatient Clinic at Asan Medical Center, South Korea. The inclusion criteria were as follows: age ≥ 18 years; a confirmed diagnosis of MS according to the McDonald Criteria (2017)^16^; provision of informed consent prior to enrollment; and absence of an acute relapse within the 2 months prior to enrollment^17–20^. Cell-based assays were performed to detect antibodies specific for aquaporin-4 protein and myelin oligodendrocyte glycoprotein: all included participants tested negative^2^. Participants were excluded if they had an acute illness (e.g., myocardial infarction, unstable angina, new onset ischemic stroke or trauma) within 2 months of the study enrollment, or active cancer^21^. The study complied with principles of the Declaration of Helsinki, and received approval from the Institutional Review Board/Ethics Committee of Asan Medical Center (No. 2018 − 0653). All participants provided written informed consent to inclusion in the study.

### Outcomes and measures

Patients’ sleep quality was assessed using the Pittsburgh Sleep Quality Index (PSQI), a self-reported survey designed to evaluate sleep quality over the past month^22^. The PSQI comprises 19 items, grouped into seven components: subjective sleep quality (C1), sleep onset latency (C2), sleep duration (C3), sleep efficiency (C4), sleep disturbance (C5), use of sleeping medication (C6), and daytime dysfunction (C7). Each component is scored on a scale from 0 to 3, with the total PSQI score ranging from 0 to 21. The PSQI has been validated extensively in various populations, including individuals with MS. This study used the Korean version of the PSQI (PSQI-K), which has been validated in the general Korean population^23^. Higher PSQI scores reflect poorer sleep quality, with a cutoff of > 5 commonly used to define poor sleep^24^. A significant worsening in sleep quality during follow-up was defined as an increase in the PSQI score by ≥ 3 points^25,26^.

HRQoL was assessed using the EQ-5D-5 L index, a self-administered survey that evaluates HRQoL across five dimensions: mobility, self-care, usual activities, pain/discomfort, and anxiety/depression^27^. Participants rated their health state on a five-level scale for each dimension, with higher levels indicating worse QoL. The EQ-5D-5 L valuation Eq. 2^8^ was applied to calculate an overall HRQoL score, represented as the EQ-5D index. Lower EQ-5D index scores reflect poorer HRQoL.

The Expanded Disability Status Scale (EDSS) was primarily assessed by MS specialist nurses and confirmed by neurologists, along with the Functional Systems (FS) Score, which evaluates symptoms or disabilities across eight neurological domains: pyramidal, cerebellar, brainstem, sensory, bowel and bladder, visual, cerebral (mental), and ambulation^29^. Follow-up EDSS scores obtained 6–12 months after baseline were used to assess disability progression. For patients with a baseline EDSS score of ≤ 5.0, an increase of at least 1.0 point, in the absence of clinical relapse at the time of confirmation, was defined as unconfirmed disability progression (UDP). For those with a baseline EDSS score ≥ 5.5, UDP was defined as a 0.5-point increase without evidence of remission. Clinical variables, including demographic data (e.g., age and sex) and disease-related information (e.g., disease duration, comorbidities, and number of relapses), were collected. Depression was defined based on ICD-10 criteria (F32–F33)^30^ and identified through corresponding diagnostic codes documented in the medical records.

### Statistical analysis

Demographic and clinical characteristics, as well as outcome variables, were summarized as percentages for categorical variables and as the median (range) for continuous variables. For longitudinal analysis, patients who experienced acute relapse during follow-up were excluded. Associations between global PSQI scores and other continuous clinical variables were examined using univariate linear regression analysis. Variables significantly associated with PSQI scores were included in multivariable regression models to identify independent predictors of both baseline and follow-up PSQI scores, adjusting for age, sex, disease duration, and baseline disability (EDSS scores). Statistical analyses were performed using IBM SPSS Statistics for Windows, version 27.0 (IBM Corp., Armonk, NY, USA). A p-value < 0.05 was considered statistically significant. Given the exploratory nature of this study, no adjustments were made for multiple comparisons.

## Results

### Patient characteristics

**Fig. 1:**
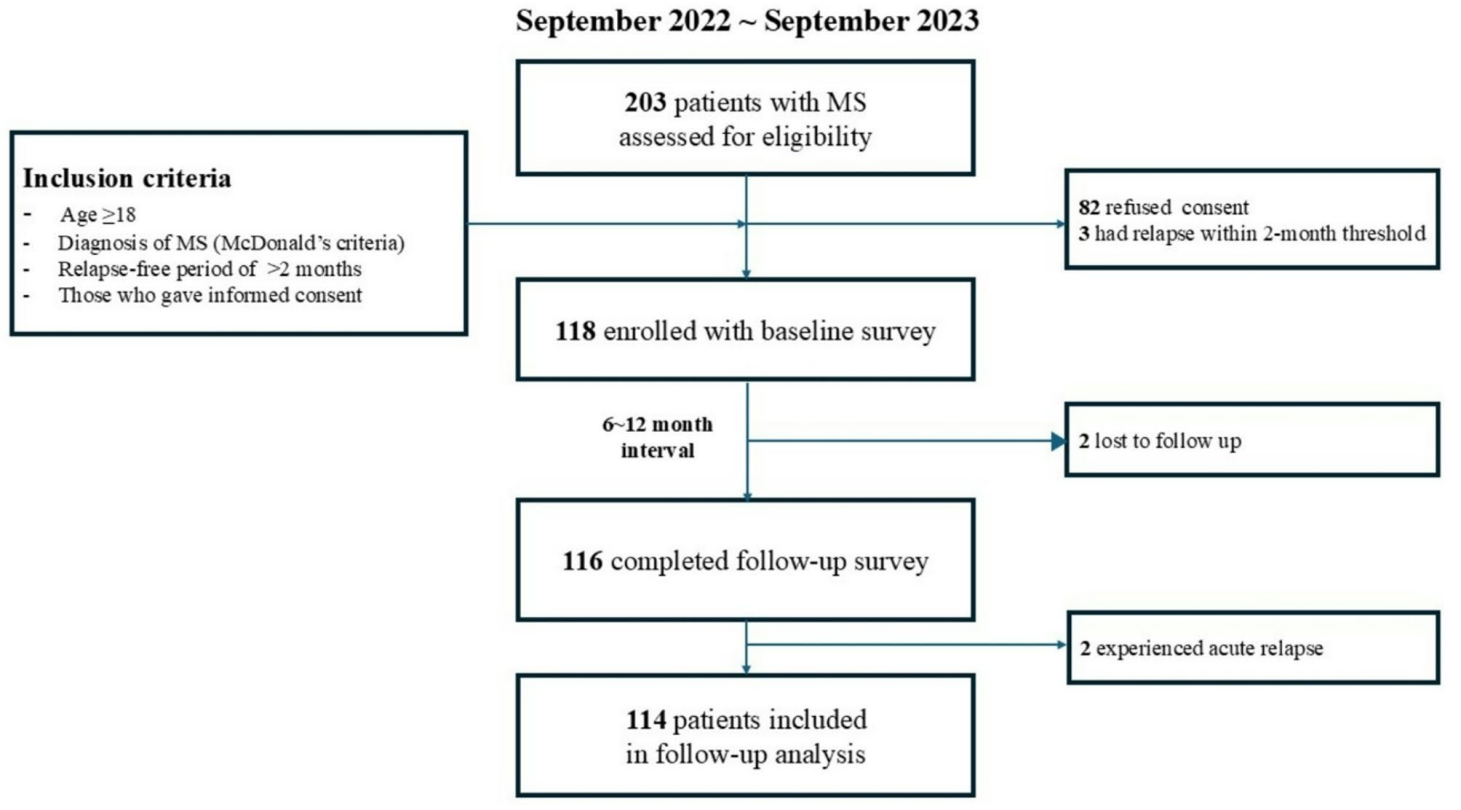
Study flow chart.

In total, 118 patients were initially enrolled and included in the cross-sectional analysis (Fig. 1). The participants had a median age of 46 years (range: 20.0–70.0; Table 1), and 71% were female. The median disease duration was 5.5 years (range: 1.0–34.0). The median EDSS score was 2.0 (range: 0‒7.5), and most (*n* = 104, 96.6%) patients were receiving disease-modifying therapies, including 10 patients receiving high-efficacy treatments (8.5%). The majority of participants (*n* = 113, 95.8%) were diagnosed with relapsing-remitting MS, four (3.4%) with secondary progressive MS, and one with primary progressive MS.

**Table 1:** Baseline characteristics of the participants, and their PSQI scores.

Characteristics	All patients	Good sleepers (PSQI ≤ 5)	Poor sleepers (PSQI > 5)	p*
	(*n* = 118)	(*n* = 54)	(*n* = 64)	
Age, y (median (range))	46.0 (20.0–77.0)	43.0 (20.0–74.0)	47.0 (25.0–77.0)	0.297
Onset age, y (median (range))	38.0 (11.0–71.0)	31.0 (11.0–67.0)	38.0 (18.0–71.0)	0.392
Female (%)	84 (71.2)	40 (74.1)	44 (68.8)	0.530
Disease duration, y (median (range))	5.5 (1.0–34.0)	6.0 (1.0–34.0)	11.0 (1.0–26.0)	0.322
Time since last attack, y (median (range))	4.4 (0.7–20.9)	4.4 (1.1–20.0)	4.7 (0.7–29.0)	0.631
Number of relapses (median (range))	1.0 (1.0–17.0)	1.0 (0.0–8.0)	2.0 (1.0–17.0)	0.010
Annualized Relapse Rate**	0.2	0.2	0.3	
Receiving DMT (n (%))	104 (96.6)	49 (90.7)	55 (85.9)	0.630
Receiving high-efficacy DMTs***	10 (8.5)	3 (5.6)	7 (10.9)	0.297
Baseline EDSS (median (range))	2.0 (0.0–7.5)	2.0 (0.0–7.5)	2.0 (0.0–7.5)	0.149
FS score: Optic Function	1.0 (0.0–6.0)	1.0 (0.0–5.0)	2.0 (0.0–6.0)	0.043
FS score: Pyramidal Function	0.0 (0.0–2.0)	0.0 (0.0–1.0)	0.0 (0.0–2.0)	0.413
FS score: Brainstem Function	0.0 (0.0–3.0)	0.0 (0.0–3.0)	0.0 (0.0–3.0)	0.717
FS score: Cerebellar Function	0.0 (0.0–4.0)	0.0 (0.0–3.0)	0.0 (0.0–4.0)	0.423
FS score: Sensory Function	0.0 (0.0–4.0)	0.0 (0.0–3.0)	0.0 (0.0–4.0)	0.247
FS score: Bowel and Bladder function	0.0 (0.0–3.0)	0.0 (0.0–3.0)	0.0 (0.0–3.0)	0.863
FS score: Cerebral Functions	0.0 (0.0–4.0)	0.0 (0.0–2.0)	1.0 (0.0–4.0)	0.025
Ambulation	0.0 (0.0–11.0)	0.0 (0.0–10.0)	0.0 (0.–11.0)	0.644
Comorbidities				
**Depression**	**11 (9.3)**	**2 (3.7)**	**9 (14.1)**	**0.054**
Systemic autoimmune disorders†	7 (5.9)	3 (5.6)	4 (6.3)	0.874
Hypertension	11 (9.3)	7 (13.0)	4 (6.3)	0.204
Diabetes mellitus	14 (11.9)	8 (14.8)	6 (9.4)	0.319
Dyslipidemia	28 (23.7)	12 (22.2)	16 (25.0)	0.818
Migraine	10 (8.5)	4 (7.4)	6 (9.4)	0.702
Epilepsy	4 (3.4)	1 (3.7)	3 (4.7)	0.399
History of cerebrovascular stroke	4 (3.4)	2 (3.7)	2 (3.1)	0.863
Baseline PSQI score (median (range))	6.0 (1.0–17.0)	4.0 (1.0–5.0)	9.0 (6.0–17.0)	< 0.001

Notes: Values are presented as number (%) for sex and type of disease-modifying therapy, and as the median (range) for EDSS and all other measures. Higher PSQI scores indicate worse sleep quality.

p*: Good sleepers vs. Poor sleepers.

SD: Standard deviation; EDSS: Expanded Disability Status Scale; PSQI: Pittsburgh Sleep Quality Index; DMT: Disease-Modifying Therapy.

**ARR: annualized relapse rate = (total number of relapses)/(total people years). Calculated based on total number of attacks since disease onset and the sum of disease duration.

***High-efficacy DMTs refer to Fingolimod, Alemtuzumab, and Cladribine.

†Systemic autoimmune conditions include autoimmune thyroid disorders (*n* = 5), systemic lupus erythematosus (*n* = 1), and ulcerative colitis (*n* = 1).

### Sleep quality and its determinants

At baseline, the median PSQI score was 6.0 (range 1.0–17.0), and 54.2% (*n* = 64) of patients were classified as poor sleepers (PSQI > 5; Table 1). Age, sex, disease duration, EDSS score, and use of disease-modifying therapies did not differ significantly between the groups; however, poor sleepers had a higher number of relapses and worse functional system scores for the optic and cerebral domains than good sleepers. According to survey results, 22.4% (*n* = 27) of patients were using sleep medications, among whom 26 were classified as poor sleepers at baseline.

Univariable analysis of factors associated with baseline PSQI scores identified significant correlations between poorer sleep quality and higher baseline FS scores (worse function) in both the optic and cerebral domains, and the presence of depression (Table 2). Multivariable analysis identified FS scores in the optic domain and the presence of depression as being associated with sleep quality. Specifically, each one-point increase in the optic FS score was associated with a 0.226-point increase in baseline PSQI scores (*p* = 0.042), while the presence of depression corresponded to a 0.261-point increase (*p* = 0.005). Although a one-point increase in the cerebral FS score was correlated to a 0.164-point increase, the results were statistically insignificant (*p* = 0.085).

**Table 2:** Correlates (predictors) of baseline PSQI scores.

	Baseline PSQI			
	Univariate Analysis	*p*	Multivariable analysis	*p*
	β (95% CI)		β (95% CI)	
Age	0.085 (−0.026, 0.070)	0.365	0.051 (−0.039, 0.065)	0.549
Sex (female)	−0.063 (−0.988, 2.001)	0.484	0.101 (−0.677, 2.308)	0.563
Onset age	0.073 (−0.029, 0.066)	0.436	–	
Disease duration (yrs)	0.021 (−0.083, 0.105)	0.821	−0.054 (−0.139, 0.083)	0.505
Time since last attack (yrs)	0.047 (−0.118, 0.185)	0.664	–	
Number of past attacks	0.103 (−0.133, 0.472)	0.270	0.114 (−0.165, 0.543)	0.178
baseline EDSS	0.065 (−0.239, 0.490)	0.496	−0.159 (−0.772, 0.159)	0.173
FSS: Optic Function	0.230 (0.126, 1.032)	0.013	0.226 (0.021, 1.115)	0.042
FSS: Pyramidal Function	−0.023 (−1.914, 1.492)	0.806	–	
FSS: Brainstem Function	−0.096 (−1174, 0.376)	0.310	–	
FSS: Cerebellar Function	−0.01 (−0.703, 0.631)	0.915	–	
FSS: Sensory Function	0.019 (−0.553, 0.675)	0.845	–	
FSS: Bowel and Bladder function	−0.054 (−1.384, 0.765)	0.569	–	
FSS: Cerebral Functions	0.201 (0.114, 2.269)	0.030	0.164 (−0.132, 2.028)	0.085
Ambulation	−0.001 (−0.274, 0.273)	0.996	–	
Depression	0.281 (1.317, 5.757)	0.002	0.261 (1.031, 5.822)	0.005

Notes: Higher PSQI scores indicate worse sleep quality.

Higher EDSS and FS scores indicate worse neurologic disability and function, respectively.

PSQI: Pittsburgh Sleep Quality Index; CI: Confidence interval; EDSS: Expanded Disability Status Scale; FSS: Functional Systems Score; β: Standardized beta coefficients.

### Sleep and quality of life

To assess the importance of sleep quality for PwMS, we examined its impact on QoL. Poor sleepers had significantly lower overall scores (median: 0.5 vs. 0.6 for good sleepers; *p* < 0.001), where lower values reflect worse QoL (Supplementary Table 1). The difference was particularly notable in the mobility (median: 2.0 vs. 1.0; *p* = 0.003) and pain/discomfort (median: 3.0 vs. 2.0; *p* < 0.001) domains, for which higher scores indicate greater impairment.

Multivariable analyses supported these findings, indicating that both poor sleep status and worse sleep quality, as reflected by higher baseline PSQI scores, were associated independently with reduced QoL (Table 3). After controlling for age, sex, disease duration, number of previous relapses, and baseline EDSS scores, poor sleep quality (PSQI > 5) remained a significant independent predictor of lower EQ-5D-5 L scores (β = −0.199; *p* = 0.032; Table 3, MODEL 1). Similarly, higher PSQI scores were significantly associated with worse QoL (β = −0.286; *p* = 0.001; Table 3, MODEL 2).

**Table 3:** Models for quality of life.

	Univariate analysis	*p*	Model 1	*p*	Model 2	*p*
	β (95% CI)		β (95% CI)		β (95% CI)	
Age (yrs)	−0.300 (−0.005, −0.001)	0.001	−0.213 (−0.004, 0.000)	0.034	−0.203 (−0.004, 0.000)	0.037
Sex (female)	0.178 (−0.001, 0.117)	0.056	0.114 (−0.020, 0.094)	0.205	0.115 (−0.018, 0.092)	0.189
Disease duration (yrs)	−0.164 (−0.006, 0.000)	0.078	0.020 (−0.004, 0.005)	0.849	0.025 (−0.004, 0.005)	0.812
Number of attacks	−0.197 (−0.025, −0.001)	0.034	−0.161 (−0.024, 0.003)	0.134	−0.176 (−0.024, 0.002)	0.087
Baseline EDSS	−0.214 (−0.031, −0.002)	0.023	−0.112 (−0.024, 0.007)	0.262	−0.123 (−0.024, 0.005)	0.201
Sleep variables						
Poor sleepers at baseline	−0.273 (−0.134, −0.028)	0.003	−0.199 (−0.111, −0.005)	0.032		
Baseline PSQI score	−0.349 (−0.021, −0.007)	< 0.001			−0.286 (−0.018, −0.005)	0.001

Notes: Higher PSQI scores indicate worse sleep quality. Higher EDSS and FS scores indicate worse neurologic disability.

PSQI: Pittsburgh Sleep Quality Index; CI: Confidence interval; EDSS: Expanded Disability Status Scale; FSS: Functional Systems Score; β: Standardized beta coefficient.

### Longitudinal sleep quality trajectories and associated factors

During the follow-up period (median: 9.2 months; range: 6.0–12.5 months), two patients were lost to follow-up and two experienced acute relapse, leaving 114 patients eligible for longitudinal analysis (Fig. 1). Among these, six patients (5.3%) exhibited UDP.

**Fig. 2:**
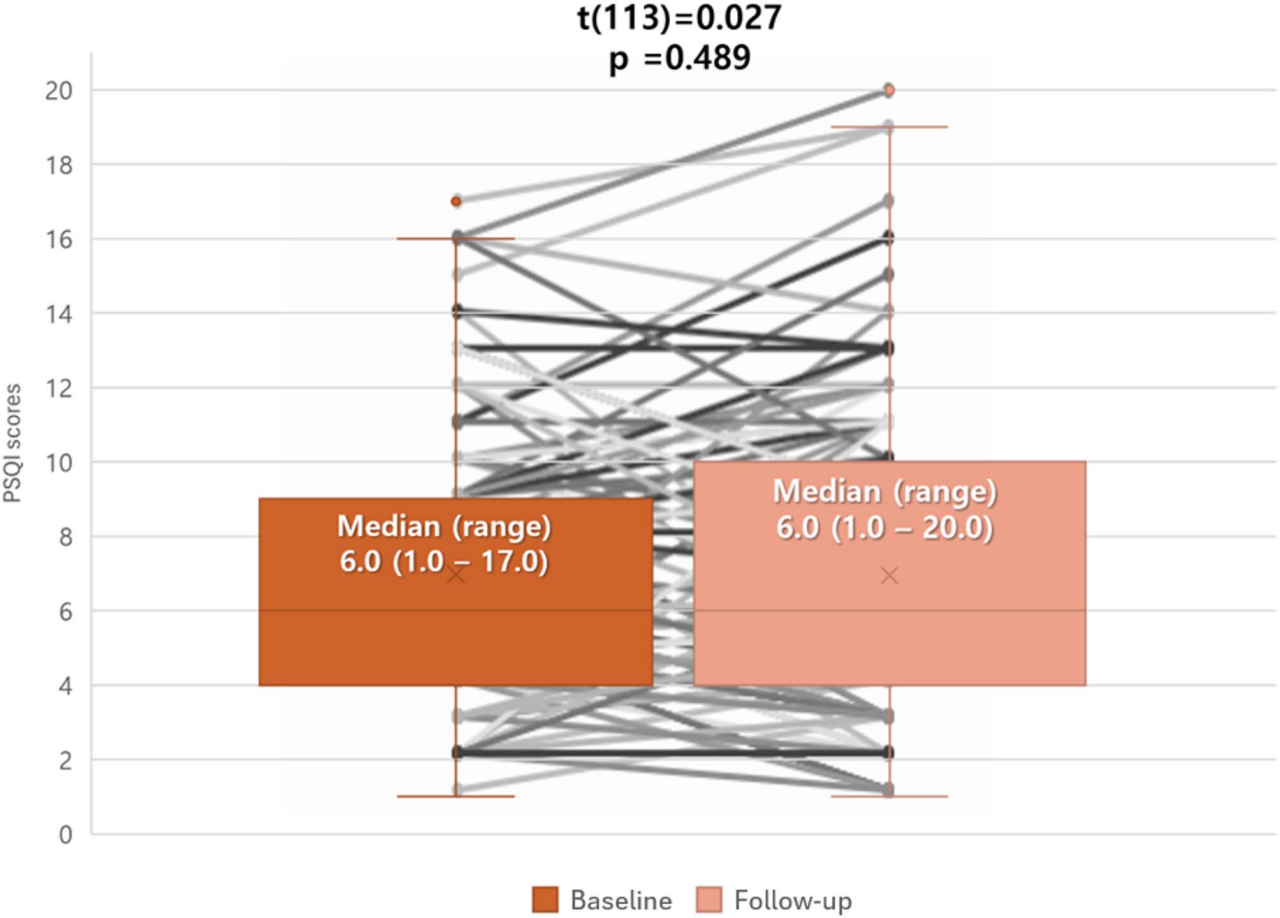
Longitudinal course of sleep quality, and changes in individuals.

At the population level, sleep quality remained largely stable over time. There was no significant change in PSQI scores from baseline (median PSQI scores: 6.0 vs. 6.0; *p* = 0.437; Fig. 2), and 53.5% (*n* = 61) of patients continued to meet the criteria for poor sleep at follow-up.

The box and whisker graph (orange bars) shows the distribution of baseline and follow-up PSQI scores in our cohort, and the gray paired line graph visualizes individual shifts in PSQI scores throughout the study period. According to paired t-test results, sleep quality remained relatively stable (unchanged) at the population level (paired t-test: p-value = 0.489).

When exploring clinical factors associated with sleep quality, univariable analysis revealed that baseline cerebral FS scores, optic FS scores, and the presence of depression were significantly associated with follow-up PSQI scores, mirroring baseline findings. In multivariable analysis, optic FS scores and depression remained significant independent predictors of follow-up sleep quality (Table 4), highlighting the stability of clinical correlates over time. There were no significant differences in PSQI scores between patients with and without UDP, and only one UDP case exhibited a substantial decline in sleep quality (Supplementary Table 2).

**Table 4:** Predictors of future PSQI scores.

	Follow-up PSQI			
	Univariate Analysis	*p*	Multivariable analysis	*p*
	β (95% CI)		β (95% CI)	
Age	0.078 (−0.032, 0.079)	0.408	0.026 (−0.051, 0.066)	0.798
Sex (female)	−0.156 (−0.252, 3.199)	0.094	−0.177 (−3.311, 0.041)	0.056
Onset age	0.013 (−0.051, 0.059)	0.894	–	
Disease duration (yrs)	0.132 (−0.031, 0.187)	0.157	0.036 (−0.102, 0.144)	0.335
Time since last attack (yrs)	0.111 (−0.087, 0.276)	0.305	–	
Number of past attacks	0.109 (−0.145, 0.560)	0.246	0.095 (−0.211, 0.572)	0.363
baseline EDSS	0.163 (−0.051, 0.793)	0.084	−0.090 (−0.728, 0.325)	0.450
FSS: Optic Function	0.236 (0.164, 1.227)	0.011	0.233 (0.038, 1.311)	0.038
FSS: Pyramidal Function	0.072 (−1.230, 2.754)	0.450	–	
FSS: Brainstem Function	0.01 (−0.863, 0.963)	0.914	–	
FSS: Cerebellar Function	0.086 (−0.423, 1.136)	0.367	–	
FSS: Sensory Function	0.043 (−0.555, 0.883)	0.652	–	
FSS: Bowel and Bladder function	−0.096 (−1.898, 0.614)	0.313	–	
*FSS: Cerebral Functions*	0.232 (0.349, 2.860)	0.013	0.170 (−0.092, 2.401)	0.069
Ambulation	0.092 (−0.163, 0.476)	0.334	–	
Depression	0.279 (1.414, 6.551)	0.003	0.215 (0.473, 5.935)	0.022

Notes: Higher PSQI scores indicate worse sleep quality.

Higher EDSS and FS scores indicate worse neurologic disability and function, respectively.

PSQI: Pittsburgh Sleep Quality Index; CI: Confidence interval; EDSS: Expanded Disability Status Scale; FSS: Functional Systems Score; β: Standardized beta coefficient.

Despite this overall stability, 23 patients (20.2%) experienced a clinically meaningful deterioration in sleep quality. This subgroup showed numerically higher cerebral and optic FS scores than the rest of the cohort, although the differences were not statistically significant (Table 5); however, multivariable logistic regression identified no independent predictors of sleep quality deterioration (Supplementary Table 3).

**Table 5:** Comparative characteristics of those with significantly worsened sleep quality (*n* = 23).

	Significantly worsened (*n* = 23)	Not worsened (*n* = 91)	*p*
Age (yrs, median(range))	48.0 (26.0–67.0)	45.0 (20.0–77.0)	0.601
Female (%)	14 (60.9%)	66 (72.5%)	0.275
Onset age (yrs, median(range))	37.0 (15.0–56.0)	34.0 (11.0–71.0)	0.641
Disease duration (yrs, median(range))	11.5 (1.0–23.0)	8.0 (1.0–34.0)	0.727
Total number of attacks (median(range))	2.0 (1.0–17.0)	2.0 (1.0–8.0)	0.424
Time since last attack (yrs, median(range))	6.3 (0.7–13.5)	4.3 (1.1–20.6)	0.682
Baseline EDSS (median(range))	2.0 (0.0–6.5)	2.0 (0.0–7.5)	0.287
FSS scores			
Optic Function	2.0 (0.0–6.0)	1.0 (0.0–6.0)	0.094
Pyramidal Function	0.0 (0.0–2.0)	0.0 (0.0–1.0)	0.971
Brainstem Function	0.0 (0.0–3.0)	0.0 (0.0–3.0)	0.404
Cerebellar Function	0.0 (0.0–3.0)	0.0 (0.0–4.0)	0.294
Sensory Function	0.0 (0.0–4.0)	0.0 (0.0–4.0)	0.732
Bowel and Bladder function	0.0 (0.0–2.0)	0.0 (0.0–3.0)	0.754
Cerebral Functions	1.0 (0.0–2.0)	0.0 (0.0–4.0)	0.067
Ambulation	0.0 (0.0–8.0)	0.0 (0.0–11.0)	0.465
Comorbidities			
Depression	2 (8.7)	9 (9.9)	0.862
Systemic autoimmune disorders†	3 (13.0)	4 (4.4)	0.134
Hypertension	0 (0.0)	11 (12.1)	0.132
Diabetes Mellitus	2 (8.7)	12 (13.2)	0.590
Dyslipidemia	7 (30.4)	21 (23.1)	0.410
Migraine	2 (8.7)	7 (7.7)	0.873
Epilepsy	2 (8.7)	2 (2.2)	0.130
History of cerebrovascular stroke	2 (8.7)	2 (2.2)	0.130

Notes: Higher PSQI scores indicate worse sleep quality. Higher EDSS and FS scores indicate worse neurologic disability and function, respectively.

PSQI: Pittsburgh Sleep Quality Index; CI: Confidence interval; EDSS: Expanded Disability Status Scale; FSS: Functional Systems Score; β: Pearson’s coefficient.

Notably, two patients who experienced relapses, but were excluded from the longitudinal analyses, also showed sleep quality deterioration. One patient with transverse myelitis (EDSS: 5.5 → 6.5) exhibited a marked increase in the PSQI score (11 → 17), while the other with optic neuritis (EDSS: 0 → 1.5) showed only a slight increase (4 → 5).

## Discussion

This prospective longitudinal study explored sleep quality and its longitudinal dynamics in PwMS. Poor sleep quality, which is significantly associated with QoL, was observed in over half of participants both at baseline and at follow-up. Although sleep quality remained largely stable at the population-level over time, there was a notable deterioration in 20.2% of patients. Baseline neurological function and the psychiatric comorbidities were found to be associated with sleep quality, with baseline optic FS scores and the presence of depression showing independent correlation with both baseline and follow-up PSQI scores.

Our findings confirm the high prevalence of poor sleep quality in PwMS, with 54% classified as poor sleepers (PSQI > 5) at baseline; these findings align with several studies reporting poor sleep in over half of PwMS^31^. The mean PSQI score in our cohort was 7.0 (SD 3.7), indicating more severe sleep quality impairment than in both the Korean general population (mean 5.6, SD 3.2)^19^ and patients with rheumatoid arthritis (mean 5.6, SD 4.2)^32,33^.

Sleep quality was identified as an independent contributor to QoL, emphasizing its clinical significance. Among QoL domains, the “Pain and Discomfort” dimension of the EQ-5D-5 L showed the strongest correlation with PSQI scores, supporting a reciprocal relationship: Poor sleep heightens pain perception^34–36^while chronic pain in turn disrupts sleep architecture^34,37,38^. The link between poor sleep quality and reduced QoL in our cohort aligns with prior evidence that associates poor sleep with increased fatigue^39^cognitive impairment^14^and overall functional decline in MS.

Longitudinally, over a median follow-up period of 9.2 months (range: 6.0–12.5 months), sleep quality remained relatively stable at the population level, with the proportion of poor sleepers showing minimal change (54.3% at baseline vs. 53.4% at follow-up). This aligns with the BETASLEEP study^40^a 2-year prospective investigation of German PwMS treated with interferon beta-1β. This study reported stable mean PSQI scores across time points (baseline (mean, SD): 6.8 (4.0); year 1 = 6.0 (3.2); year 2 = 6.3 (3.6)) and similar proportions of poor sleepers over the follow-up period (57.1% at baseline and year 1, and 53.6% at year 2). Given the significant impact of poor sleep on the QoL of PwMS, these findings underscore the importance of early identification and long-term management of sleep disturbances in this population.

Optic dysfunction emerged as an independent risk factor for poor sleep at both baseline and follow-up. Optic neuritis, a common manifestation of MS, leads to demyelination of the optic nerve and impaired light transmission^2^. Given that circadian rhythms are regulated by photic input from the retina to the suprachiasmatic nucleus^41^damage to the visual pathway may disrupt circadian rhythm entrainment, thereby contributing to generally reduced sleep quality^42,43^. This mechanism may explain the association between optic dysfunction and elevated PSQI scores observed in our cohort. Although this finding emphasizes the role of insomnia-like symptoms, it does not diminish the potential contribution of other sleep disorders such as obstructive sleep apnea (OSA) or restless legs syndrome (RLS). It should be noted that the PSQI is particularly sensitive to symptoms typical of insomnia, such as prolonged sleep latency and poor sleep efficiency, while being less effective at capturing disturbances more specific to OSA or RLS, such as breathing pauses or limb movements^45^. Given that various sleep disorders are prevalent among PwMS, and we did not specifically assess their presence, the observed poor sleep quality should not be attributed solely to insomnia-like symptoms.

Depression was identified as another independent risk factor for poor sleep quality in PwMS, consistent with existing literature highlighting the strong association between mental health and sleep disturbances. Depression is highly prevalent in MS and may contribute to sleep disruption through several mechanisms, including neuroinflammation, hypothalamic–pituitary–adrenal (HPA) axis dysregulation, and altered monoaminergic signaling^46^. Clinically, depressive symptoms such as difficulty initiating or maintaining sleep and non-restorative sleep are well reflected in elevated PSQI scores^47^ Furthermore, depression often coexists with fatigue, which may amplify perceived sleep disturbances and further deteriorate sleep quality^4,48–50^. Our findings suggest that the adverse impact of depression on sleep is not transient but may persist throughout the disease course, reinforcing the need for routine screening and appropriate management of psychiatric symptoms in PwMS to improve both mental health and sleep-related outcomes.

While cerebral FS scores initially appeared to correlate with sleep quality, this association did not remain significant after adjusting for other covariates in multivariate analyses. Cerebral dysfunction, including cognitive impairment and mood disturbances, is linked closely to sleep disruption and may interact bidirectionally, with poor sleep quality potentially exacerbating these symptoms^7,15^. Notably, patients who experienced a clinically meaningful decline in sleep quality over time had higher baseline cerebral and optic FS scores, although these factors were not identified as independent predictors in the multivariable regression. Future larger studies are warranted to confirm these associations.

This study has several strengths. Unlike many cross-sectional investigations, we collected longitudinal data, capturing changes in sleep quality at the individual level over time. Another key strength is inclusion of detailed neurological evaluations, combining FS scores with EDSS. While the EDSS is used widely, its emphasis on ambulation and visual function may underrepresent cognitive and affective aspects of disease burden^51^. By integrating FS scores, our approach offers a more comprehensive assessment of neurological status and its association with sleep quality. Importantly, our findings suggest that baseline optic dysfunction (and to some degree, cerebral dysfunction) may serve as early indicators of future sleep quality. Identifying such predictors is clinically relevant, as sleep is modifiable, and linked closely to QoL and disease progression in PwMS.

Several limitations should be acknowledged. First, this single-center study involved a relatively small sample size, which May limit the generalizability of the findings. Second, the follow-up period of 6–12 months provides only a short observational window, potentially limiting detection of long-term sleep trajectories. Our method of assessing sleep quality, the PSQI questionnaire, has several important considerations. Although the PSQI is widely used to evaluate overall subjective sleep quality, elevated scores may arise from a variety of underlying factors, including insomnia symptoms, circadian rhythm disruption, nocturnal pain, or unrecognized comorbid conditions such as OSA or RLS. The PSQI does not differentiate between these contributing factors and is primarily validated for identifying insomnia-like symptoms. As such, it may not accurately capture disturbances specific to disorders like OSA^45^. Since we did not perform polysomonography, assess specific types of sleep disturbances, or administer disorder-specific screening tools (e.g., STOP-Bang for OSA^52^ RLS-DI for RLS^53^), elevated PSQI scores in our cohort should be interpreted as a general reflection of poor subjective sleep quality rather than as indicative of a particular sleep disorder. This represents a limitation of the present analysis and highlights the need for more comprehensive sleep assessments in future studies. Regarding our data on depression, our analysis of psychiatric disorders (with the use of diagnostic codes) may also have underestimated the presence of depressive symptoms. Use of psychiatric surveys such as the Beck’s Depression Inventory (BDI) should be included in future longitudinal studies to further elucidate long term associations between sleep and depressive symptoms. Additionally, the impact of disease progression on sleep was not directly assessed, particularly in terms of subclinical disease activity, including chronic lesion burden, brain and lesion volumes on imaging, and serum biomarkers such as neurofilament light chain. Future studies should aim to include larger and more diverse cohorts, extend the follow-up duration, and incorporate imaging and biological markers to more accurately characterize the longitudinal relationship between MS progression and sleep quality.

## Conclusion

In conclusion, our study demonstrates that poor sleep quality is highly prevalent among PwMS and is significantly associated with reduced quality of life. Although sleep quality tended to remain stable over time, a considerable proportion of patients experienced further deterioration. The strong associations between poor sleep and neurological factors, particularly optic dysfunction and depression, underscore the need for personalized sleep management strategies.

## Supplementary Information

Below is the link to the electronic supplementary material.

Supplementary Material 1

## Data Availability

The datasets used and/or analysed during the current study available from the corresponding author or the first author (Habin Yun, mail : habin0330@gmail.com) on reasonable request.
