# Supplementary material for: Longitudinal changes in sleep quality, and their predictors in patients with multiple sclerosis
Source: Sci Rep. 2025 Sep 26;15:33153. doi: 10.1038/s41598-025-18693-5 (PMC12475199; doi:10.1038/s41598-025-18693-5)
Supplement: Supplementary file 1 — Supplementary Material 1 [file 41598_2025_18693_MOESM1_ESM.docx]

**Supplementary table 1. Baseline quality of life measures**

|  | All patients | Good sleepers (PSQI <5) | Poor sleepers (PSQI ≥5) | p* |
| --- | --- | --- | --- | --- |
| Baseline EQ-5D-5L index score* (median (range)) | 0.504 (0.006–0.592) | 0.546 (0.115–0.592) | 0.451 (0.006–0.592) | <0.001 |
|  |  |  |  |  |
| EQ-5D-5L component scores** (median (range)) |  |  |  |  |
| Mobility | 1.0 (1.0–5.0) | 1.0 (1.0–5.0) | 2.0 (1.0–5.0) | 0.003 |
| Self-care | 1.0 (1.0–4.0) | 1.0 (1.0–4.0) | 1.0 (1.0–4.0) | 0.057 |
| Usual activities | 1.0 (1.0–5.0) | 1.0 (1.0–4.0) | 1.0 (1.0–5.0) | 0.126 |
| Pain and discomfort | 2.0 (1.0–5.0) | 1.0 (1.0–4.0) | 3.0 (1.0–5.0) | <0.001 |
| Anxiety and depression | 2.0 (1.0–5.0) | 1.0 (1.0–4.0) | 2.0 (1.0–5.0) | 0.105 |

**Notes)** Lower EQ-5D-5L index scores (range : 0–1) indicate worse quality of life,
Higher component scores (1-5 Likert scale) indicate worse quality of life in each dimension.

p*: Good sleepers vs. Poor sleepers

**Abbreviations:** PSQI: Pittsburgh Sleep Quality Index

**Supplementary table 2. Sleep quality in those with unconfirmed disability progression**

|  | Undetermined disability progression* | No Disability progression |  |
| --- | --- | --- | --- |
| Comorbidities | (n = 6) | (n = 108) | p |
| Baseline PSQI (mean (range)) | 8.0 (2.0–13.0) | 6.0 (1.0–17.0) | 0.304 |
| Baseline poor sleepers (n (%)) | 5 (8.3%) | 57 (52.8%) | 0.144 |
| Follow-up PSQI (mean (range)) | 8.0 (2.0–11.0) | 6.0 (1.0–20.0) | 0.853 |
| Follow-up poor sleepers (n (%)) | 4 (66.7%) | 57 (52.8%) | 0.507 |
| Significant worsening of sleep quality (n (%)) | 1 (16.7%) | 22 (20.4%) | 0.826 |

*Undetermined disability progression was defined as follows: for patients with a baseline EDSS score ≤5.0, an EDSS score increase of at least 1.0 point from baseline, with no relapse at the time of confirmation. For patients with a higher baseline EDSS (≥5.5), a 0.5-point increase threshold was used.

**Supplementary table 3. Risk factors for significant worsening of sleep quality**

|  | Multivariate analysis |  |
| --- | --- | --- |
|  | β (95% CI) | p |
| Age | 0.991 (0.955, 1.028) | 0.892 |
| Sex (female) | 0.543 (0.198, 1.486) | 0.181 |
| Onset age | – |  |
| Disease duration (yrs) | 1.019 (0.941, 1.104) | 0.743 |
| Time since last attack (yrs) | – |  |
| Number of past attacks | 0.904 (0.655, 1.248) | 0.611 |
| baseline EDSS | 1.023 (0.741, 1.414) | 0.761 |
| FSS: Optic function | 1.272 (0.888, 1.822) | 0.118 |
| FSS: Pyramidal function | – |  |
| FSS: Brainstem function | – |  |
| FSS: Cerebellar function | – |  |
| FSS: Sensory function | – |  |
| FSS: Bowel and bladder function | – |  |
| FSS: Cerebral functions | 1.607 (0.785, 3.291) | 0.199 |
| Ambulation | – |  |

To examine whether cerebral and optic FS scores could predict worsening of sleep quality, we made a logistic regression model including baseline cerebral and optic FS scores, and controlled for the effects of age, sex, disease duration, number of attacks, and baseline EDSS scores. Neither variable showed significant results.
